# A Wireless and Batteryless Microsystem with Implantable Grid Electrode/3-Dimensional Probe Array for ECoG and Extracellular Neural Recording in Rats

**DOI:** 10.3390/s130404624

**Published:** 2013-04-08

**Authors:** Chih-Wei Chang, Jin-Chern Chiou

**Affiliations:** 1 Department of Bioengineering, University of California, Los Angeles, CA 90095, USA; E-Mail: cw.chang@ucla.edu; 2 Department of Electrical and Computer Engineering, National Chiao-Tung University, 1001 Ta Hseuh Rd., Hsinchu City 30010, Taiwan; 3 Department of Medicine, China Medical University, No.91, Hsueh-Shih Road, Taichung City 40402, Taiwan

**Keywords:** microsystem, wireless, neural electrode array, biopotentials

## Abstract

This paper presents the design and implementation of an integrated wireless microsystem platform that provides the possibility to support versatile implantable neural sensing devices in free laboratory rats. Inductive coupled coils with low dropout regulator design allows true long-term recording without limitation of battery capacity. A 16-channel analog front end chip located on the headstage is designed for high channel account neural signal conditioning with low current consumption and noise. Two types of implantable electrodes including grid electrode and 3D probe array are also presented for brain surface recording and 3D biopotential acquisition in the implanted target volume of tissue. The overall system consumes less than 20 mA with small form factor, 3.9 × 3.9 cm^2^ mainboard and 1.8 × 3.4 cm^2^ headstage, is packaged into a backpack for rats. Practical *in vivo* recordings including auditory response, brain resection tissue and PZT-induced seizures recording demonstrate the correct function of the proposed microsystem. Presented achievements addressed the aforementioned properties by combining MEMS neural sensors, low-power circuit designs and commercial chips into system-level integration.

## 1. Introduction

Neuroscience engineering together with biomedical devices for neural recording in the central nervous system are widely viewed as a promising path to revolutionary progress in understanding neural functions and for the realization of practical neural prostheses. Also, long-tem, real-time and stable observation of the monitoring target for treating disorders such as seizures, as well as for rehabilitative prosthesis is required for these biomedical device designs that can simultaneously record neural signals from a large number of electrodes in awake animals [1]. Due to the small size of laboratory rats, a miniature, lightweight, wireless, implantable microsystem is the key solution to capture accurate biological signals from an untethered animal in its natural habitat [2,3]. Generally, wire connections are required for powering and data transmission. Use of wire connection between animals and instrumentation can cause serious problem, and even lead to the death of implanted animals when wire drag happens. Swivels are used to solve the dragging problem, but the area of movement is still limited and the price can be another problem. Additionally, channel numbers are limited and the implanted laboratory animal still cannot behave in their nature condition because of the wires. Benefitting from rapid developments in wireless communication, various wireless solutions including Zigbee [4] and Bluetooth [5], have been commercialized and applied in animal studies. Batteries are commonly used to eliminate the problem of power wires but they dominate the size and the cost of the implant device and become less attractive when the implant devices get smaller and one needs to occasionally replace their batteries [6]. Therefore, the demand for developing continuous long-term monitoring in free-behavior implanted animals is then highly desirable.

System-level integration provides efficient and low noise performance due to the compact size and shorter interconnections. Microsystem platforms with analog front ends, micro controller units and wireless data/power transferring ability offer the possibility to connect with all kind of neural sensing elements for different applications. Such microsystems provide amplification, filtering, digitization and wireless transmission for the raw signals collected from implantable sensors in the brain, and also lengthen implant life and avoid the wire plugs and additional size/weight required [7].

The majority of this paper is dedicated to the development of a biomedical microsystem platform associated with versatile implantable electrodes, that provides wireless and battery-less data and power transmission to/from an external host. A brief description with preliminary results has been presented in [8]. Two customized chips including a multichannel analog front end and a low dropout regulator are integrated with a commercial MCU and Bluetooth into a backpacked headstage-mainboard system. Also, two types of implantable electrodes, including a grid electrode and a 3D probe array, are presented for brain surface recording and 3D biopotential acquisition in the implanted target volume of tissue. In this paper, application examples will include auditory response measurement and seizure spike-wave discharge (SWD) monitoring in rats. The microsystem addressed the aforementioned properties by combining MEMS neural sensors, low-power circuit designs and commercial chips into system-level integration.

## 2. System Structure

**Figure 1.**
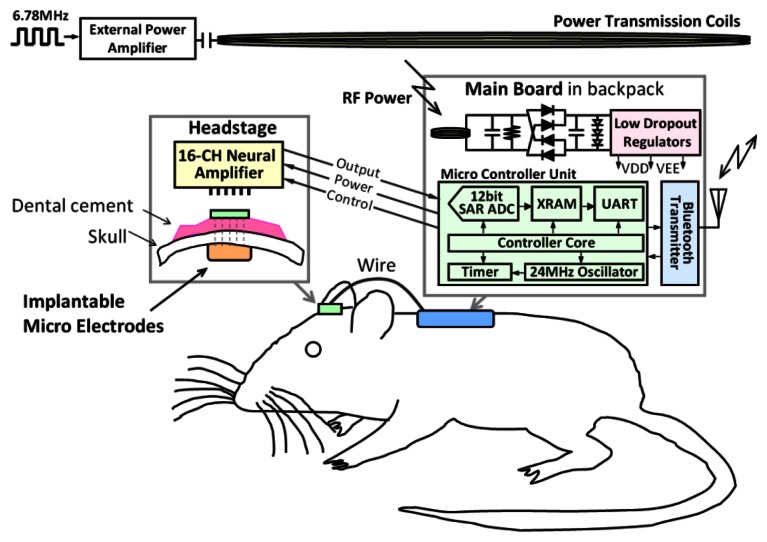
System structure of the presented microsystem.

The presented microsystem comprises three typical elements: electrodes, connection cable and signal processing circuitry [9]. Figure 1 presents the system structure design and the application situation using the head-stage and main board in a backpack on a rat. The microelectrodes are implanted into the brain, connected to a connector and encapsulated with dental cement. A headstage with a 16-channel neural amplifier is then plugged onto the connector on the rat's head. The recorded signal from the electrodes is then amplified, filtered by the analog front end and output to the main board. Wires between the headstage and main board transferring output signals from the headstage and power/control signasl from the MCU are made from EEG lead wire for less noise sensitivity. A backpack is designed for rats to carry the main board, which consists of a microcontrol unit, Bluetooth transceiver, RF power receiving coil, rectifier and low dropout regulator. The external power transmission coils are located on the cage, driven by a power amplifier and a RF signal generator.

The main power loss in inductive power transmission is the electromagnetic (EM) wave reflection at the air-skin interface and heat dissipation effect inside the tissues [10]. Moreover, according to a prior study [11], skin tissue shows high absorption and reflection properties towards EM waves when the EM frequency is higher than 20 MHz and lower than 1 MHz. In this paper, one of the industrial, scientific and medical (ISM) radio bands (6.78 MHz) is chosen to meet the requirement of low tissue absorbability to avoid tissue damage.

## 3. Implantable Sensing Devices

### 3.1. 3D Microprobe Array

**Figure 2.**
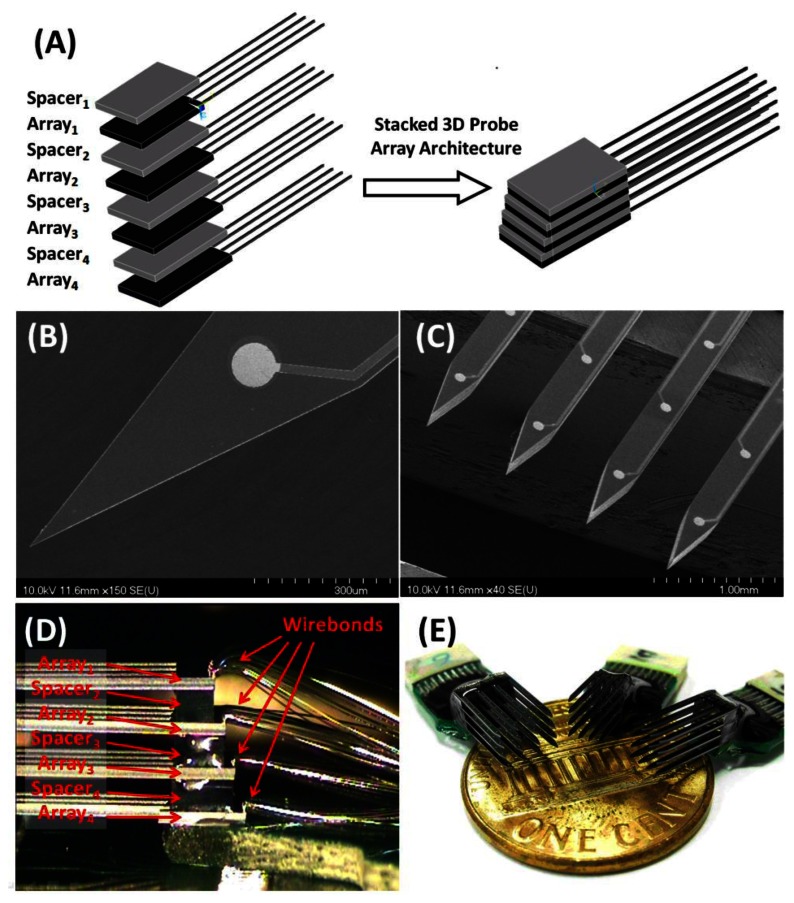
Three dimensional neural probe array. (**A**) Stacking method (**B**,**C**) Close view of the electrode site and shafts (**D**) Wire bonding from each level of the array (**E**) Assembled array on one cent coin.

To access the full cell activity that originates in the target tissue, three dimensional distributed electrode arrays are required to achieve the recording and mapping of the neural signal network in the brain structure, which would be impossible to achieve by using 2-D planar arrays [12,13]. The stacking method for three-dimensional neural probe arrays creates 3-D probe arrays by assembling 2-D arrays and spacers layer by layer. As shown in Figure 2(A), for a 4 × 4 3-D array, four 2-D arrays with four probes in each array and three spacers were required. Figure 2(B,C) shows microphotography images of the 2D arrays. Each 2-D array can be wire-bonded individually. Therefore, perpendicular bonding pads [14], folded structure with complex assembly [15,16] method are no longer needed. After the 2D parts were fabricated, a flip-chip bonder and thermosetting polymer were used in the assembly process. Convenient flip-chip technology was employed to accomplish the alignment, pressurization and heating process, while the thermosetting polymer provided an adhesive layer between two stacked layers. The thermosetting glue solidified at 185 °C in 180 s with an adhesive strength of 150–180 kg/cm^3^. The maximal placement accuracy of the flip-chip was 0.5 μm in a single bonding step. The average assembly time for a 4 × 4 3D microprobe array by manual alignment was approximately 35 minutes, including heat curing time. Figure 2(D) shows the side view of wire bonding from each level of the array. Note that the thermosetting polymer layer is hidden between stacked layers therefore may not be visible. Figure 2(E) displays a photograph of the fabricated and assembled 3-dimensional probe array consisting of 16 silicon shafts with 3D distributed platinum recording sites on a one cent coin. The recording site is 100 μm in diameter and narrow tip is about 23°.

### 3.2. Flexible Grid Electrode Array

A flexible grid electrode array designed to be placed onto the brain cortex surface is proposed in this paper and acts as an implanted sensory device providing raw ECoG signals to the wireless microsystem. The design criteria of the grid electrode array include thickness, flexibility and bio-compatibility. Microstructure thickness allows minimal implantation damage [17]. Highly flexible structure properties are required to fit the curved brain surface. The presented grid electrode array is formed by 12 μm thick Parylene-C as substrate with 500 μm diameter platinum electrodes as sensing material. Benefitting from its flexibility properties, the grid electrode array can perfectly fit the brain surface curvature and collect the neural activity signals from the cortex surface.

**Figure 3.**
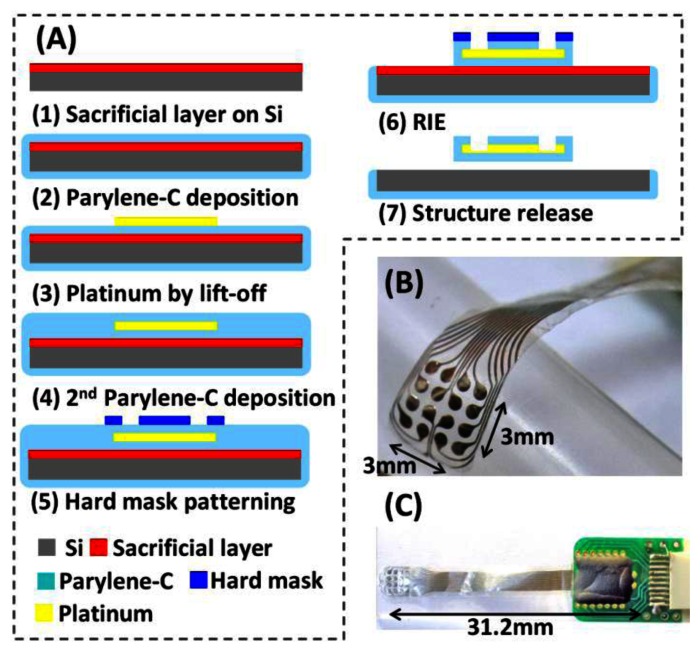
Flexible grid electrode array (**A**) Fabrication process. (**B**) Close-up view of the sensing electrodes. (**C**) Packaging and connection by a connector to the printed circuit board.

Figure 3 illustrates the proposed fabrication process flow: (1) sacrificial layer deposition on the substrate; (2) Parylene-C deposition using CVD technique; (3) platinum electrode and interconnection wire definition using lift-off technique; (4) 2nd Parylene-C deposition; (5) hard mask patterning for grid electrode array shape definition; (6) dry etching Parylene-C; (7) structure release by washing the sacrificial layer material. Figure 3(B) shows a close-up view of the recording electrode array. Parylene-C shows its transparency propertiesy in Figure 3(C). The grid electrode array is packaged on a pre-designed PCB with a connector to be linked to the headstage. The grid array is 31.2 mm in length, 4.3 mm in width, and approximately 12 μm in thickness. A total 1 of 16 platinum electrodes are fabricated with 500 μm in diameter, 750 μm in pitch. The width of the routing wire is 100 μm.

## 4. Wireless and Batteryless Microsystem

### 4.1. RF Powering Electronics

The RF-powering system block diagram is shown in the upper part of the main board in Figure 1. External RF power produced by a Class-E amplifier is coupled to the microsystem via a tuned LC network followed by a full-wave rectifier and LDO regulators to produce stable system and reference supplies. Low forward voltage Schottky diode and low equivalent series resistance (ESR) capacitance are selected for low ripple noise performance.

To date, the study of inductive coupled RF power transmitted by coils has been well established by following the electromagnetic induction of Faraday's Law [18]. Various approaches have been presented for optimal inductive coil design in implantable electronics [19–21]. In this paper, the transmission coils are designed to be located on the wall of the cage. The design procedure follows the method we used before and can also be found in [10,22–24]. The fabricated receiving and external coils made by 24/16 AWG copper wire are 1.5 cm and 22 cm in diameter. The inductive link exhibits pretty low coupling efficiency due to the large transmission coils design, but enough for powering the proposed microsystem. Efficient power coupling design in a large moving area for implanted animals will be our next step.

**Figure 4.**
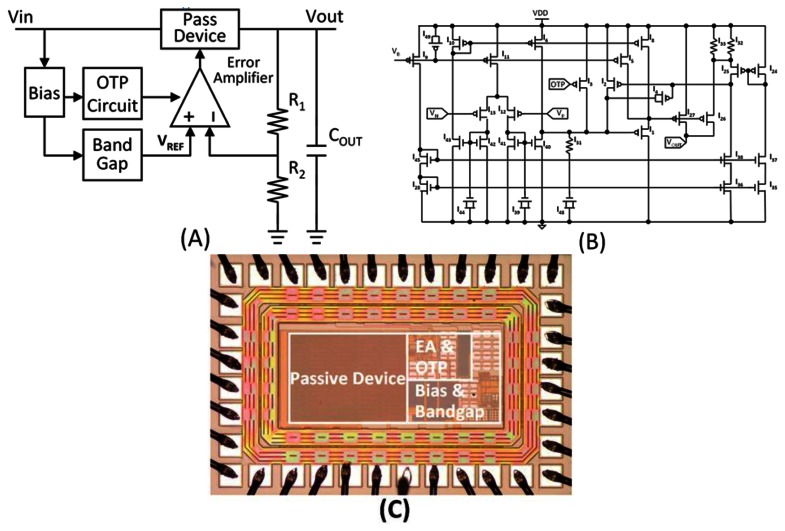
(**A**) Block diagram of the LDO regulator (**B**) Schematic of error amplifier (**C**) Microphotograph of the fabricated LDO regulator chip.

Figure 4(A) illustrates the block diagram of the low-dropout linear regulator. The architecture is modified from a typical low-dropout regulator topology with power MOSFET and OTP to enhance the driving current and avoid temperature damage to tissue [25]. The error amplifier amplifies the voltage difference between the reference voltage and divided load voltage and switches the power MOSFET (PMOS). Figure 4(B) shows the schematic of the error amplifier. MI27 is the power MOSFET illustrated in Figure 4(A). The over-temperature protection (OTP) design is used to avoid thermal damage to tissues due to the high temperature (>40 °C) caused by the operating circuit. The OTP concept is realized by ROND resistor in tsmc 0.35 μm process. Hysteresis functions when temperature is higher than 40 °C, the power MOSFET turns off; when temperature drop down lower than 37 °C, OTP pull high to open the power MOSFET again. Figure 4(C) shows the optical microphotograph of the fabricated LDO regulator chip via a TSMC 0.35 μm 2P4M process. The die size is 1.42 × 0.95 mm^2^.

### 4.2. 16-channel Analog Front End

**Figure 5.**
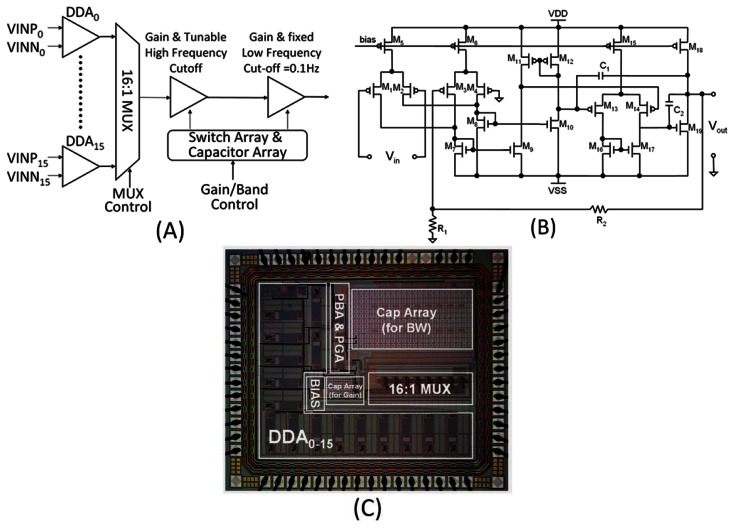
(**A**) Block diagram of the 16-channel amplifier, (**B**) Schematic of the differential difference amplifier, (**C**) Microphotograph of fabricated neural amplifier chip.

The 16-channel analog-front-end (AFE) amplifier provides signal conditioning and filtering of the weak neural potentials recorded by the electrode arrays. The block diagram of the complete 16-channel amplifier is shown in Figure 5(A). Differential difference amplifiers (DDA) are used as instrumentation stage of the AFE amplifier to meet our high-CMRR and low-noise requirements. Compared to other instrumentation amplifier designs, the DDA structure benefits from its high CMRR and device mismatches in the DDA design will solely influence the amplification gain. A 16:1 MUX controlled by the clock signal allows all 16 DDAs the share the same second and third amplifier/filter stages. The MUX frequency is set up to 200 kHz to scan the 16-channel inputs. The second stage and third stage use operational transconductance amplifier (OTA) to implement low-pass filter, high-pass filter and gain amplifier with selectable bandwidth and gain. The gain and high frequency cut-off are controlled by a switchable capacitance array. The DDA used in this work is depicted in Figure 5(B). To reduce the flicker noise, the input stages are PMOS with wide width and operated in weak inversion region. The input stage transfers the input voltage into current. Common source amplifier is then transferring the current into differential input of the following two-stage amplifier. Finally, the DDA-based non-inverting amplifier is implemented from the topological placement of R1 and R2.

The 16-channel neural amplifier is fabricated using the TSMC 0.35 μm 2P4M CMOS process. A microphotograph of the complete chip is shown in Figure 5(C). The whole chip has a size of 4.18 mm^2^, including pads.

### 4.3. Integrated Microsystem

**Figure 6.**
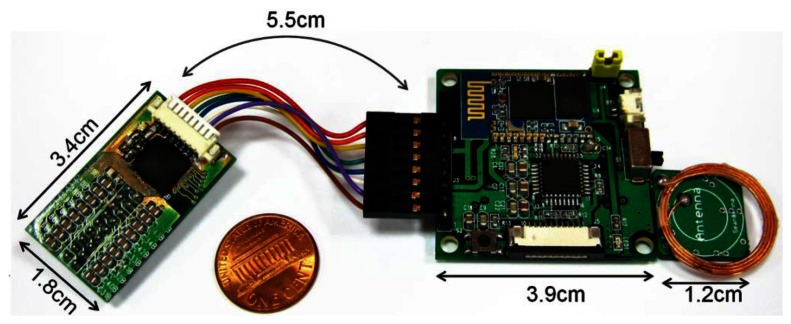
Fabricated microsystem including headstage and main board.

The fabricated AFE chip is integrated onto the headstage by Chip-on-Broad (COB) technique with other associated passive components. The headstage is 3.4 cm × 1.8 cm in size and weighs 3.92 g. A commercial controller unit, Bluetooth wireless transceiver and the RF powering sub-circuit were integrated as the main board. A 4-layer PCB design is utilized with two powering layers in the middle layers. Figure 6 shows the picture of the fabricated microsystem including the headstage and main board. Note that the RF power receiving module is designed to be attached in the backside of the main board. The LDO regulators are located on the backside of the RF power receiving module which was illustrated in [8].

The front side of the main board consists of an MCU (C8051F58, Sliconlab Inc.) and wireless Bluetooth transceiver (BTM-192, Rayson Inc.). The main board is 39 mm × 39 mm in size, and weighs 9.35 g including the RF power receiving module.

The presented LDO regulator provides 3.3 V, 1.8 V and 0.7 V for the ESD circuit, system VDD and reference bias, respectively. Also, an analog-digital DC voltage isolation technique including use of the middle two layers of the PCB as analog ground and digital ground, and magnetic beads used for connection between the analog and digital ground is utilized to isolate the digital noise coupling to the analog circuitry. Additionally, programming ports, power on LED, reset button and debugging circuitry are embedded on the board for function verification, although this can seriously increase the system dimensions. Detailed system specifications are summarized in Table 1.

The MCUis 8051 core based micro controller with embedded 200 ksps 12-bit SAR ADC, 50 MHz oscillator, operates under 1.8 V. A UART port is used to communicate with the Bluetooth module, which consists of a CSR Bluecore-4 single chip, complete 2.4 GHz radio transceiver and print circuit antenna, designed for wireless connection. Benefitting from the easy usage of Bluetooth, a single laptop computer is utilized as transceiver host.

**Figure 7.**
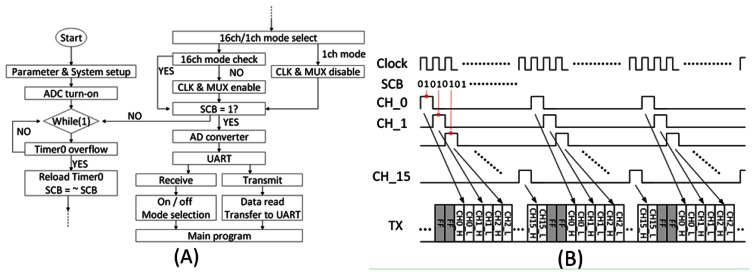
(**A**) Control flow of the MCU (**B**) Data timing and packaging method.

Figure 7(A) shows the control flow of the MCU, and detailed timing chart of the data is in Figure 7(B). The microsystem provides two operation modes, namely 16-channel mode and single channel mode. In the case of 16-channel mode, the clock signal generated by the MCU is sent to the AFE amplifier to scan the 16 channels and output the series data. A counter controlled Sample-control-Bit (SCB) is used to sample the analog data to ADC at the falling edge of the clock. Digitalized 12-bit data is then divided into first 8-bit and later 4-bit (2 bytes) because the UART only transfers 8-bit data in one time. After 16 channels are sent, two FF which acts marker are sent into UART before next 16-channel data. The marker is used by the software to divide and reconstruct the serial data back to simultaneous 16 channels. Windows-based software is designed for receiving data from the presented microsystem and sends commands to turn on, reset or parameter setting to the microsystem.

## 5. Experimental Results

**Figure 8.**
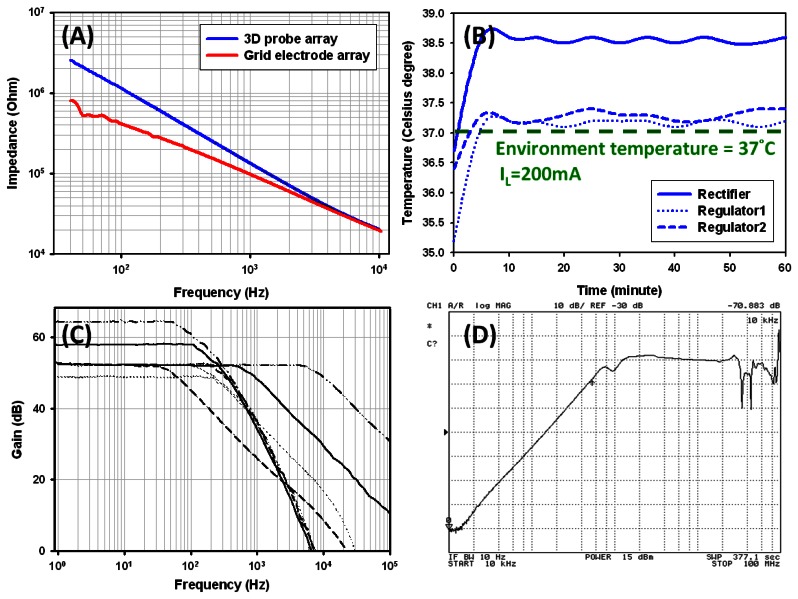
(**A**) Impedance spectrum (**B**) Temperature raise of regulators (**C**) Tunable Gain/Band (**D**) PSRR performance of the LDO regulator.

Impedance spectroscopy was used to evaluate the impedance performance of the electrode- tissue interface. When the electrode sites come into contact with tissue, electrode-tissue interface impedance was established. High interface impedance will cause signal attenuation and induce considerable thermal noise while recording. Figure 8(A) displays the impedance spectrum of the grid electrode and 3-dimensional probe array in physiological saline solution, which is used to simulate the recording conditions. Result shows about 10 kΩ impedance at 1 kHz. The grid electrodes have lower impedance due to the larger electrode area.

Figure 8(B) illustrates the temperature raise test of the wireless powering module under 37 °C environmental temperature, which is used to simulate the practical implant environmental conditions with maximal output current (200 mA) for 1 h. The powering module was put into an oven heated to 37 °C to simulate the human body temperature. As shown in Figure 8(B), the raise of chip temperature follows the oven temperature, showing that less than 2 °C raise is achieved to meet the implantation requirements [13,26]. The LDO regulator also achieves low Quiescent-Current at 45 μA and high loading current 0–200 mA capability with thermal protection. Tunable gain/band property of the 16-ch AFE amplifier is shown in Figure 8(C). Figure 8(D) is the test result of the power supply rejection ratio. The measured PSRR is around 70.883 dB at 10 kHz, indicating that it shows less PSRR than the simulation result due to parasitic inductance existing in the output ceramic capacitance, which decreases the PSRR performance.

**Figure 9.**
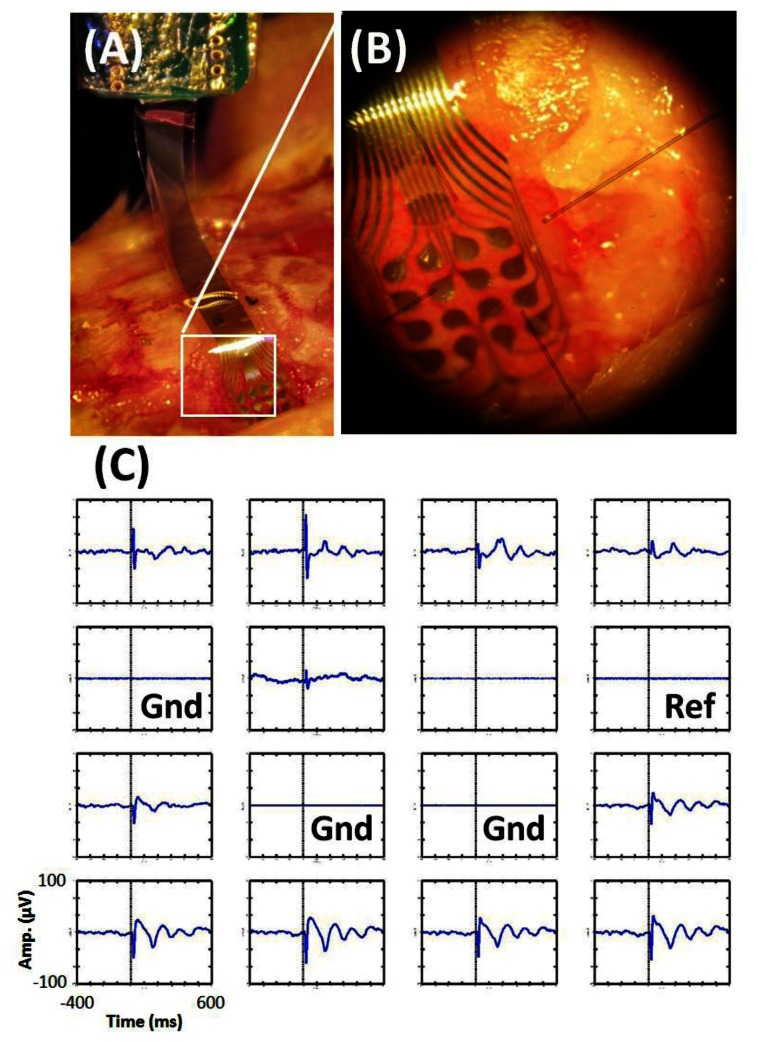
(**A**,**B**) Optical photograph of the grid electrode implantation (**C**) Time-magnitude plot of the averaged ECoG response under 9 kHz 75 dB SPL stimulation.

To demonstrate the functionality of the fabricated gird electrode array, *in vivo* experiments on auditory stimulation response recording are induced. When an anesthetized rat receives specialized frequency/magnitude sound stimulation, an electrical response can be observed on a localized auditory area of the cortex. When the sound stimulation is received by the ears, it is transformed into neural signals and sent into brain which causes neural activities appearing in the auditory sensory area. By using the flexible grid electrode array, the area of interest of the brain is covered and recorded. Therefore, a localized electrical response spot could be detected and characterized for functional mapping and event evaluation. Practical implantation optical photograph is presented in Figure 9(A), which shows how the flexibility of the system allows excellent fitting to the exposed brain cortex. Figure 9(B) shows the close-up view of the electrode array contact area.

Figure 9(C) shows the auditory response of rat recorded by the grid electrode array with 9 kHz sound stimulation. Measured results shows that localized neurons discharge phenomenon (gamma activities, 30–200 Hz) are recorded by the presented grid electrode array. Three different auditory areas are covered by the grid electrode due to the different pattern observations. The experimental results show that recorded evoked potential on auditory cortex is about ±70 uV, and the minimal effective sound stimulation magnitude is 20 dB SPL.

**Figure 10.**
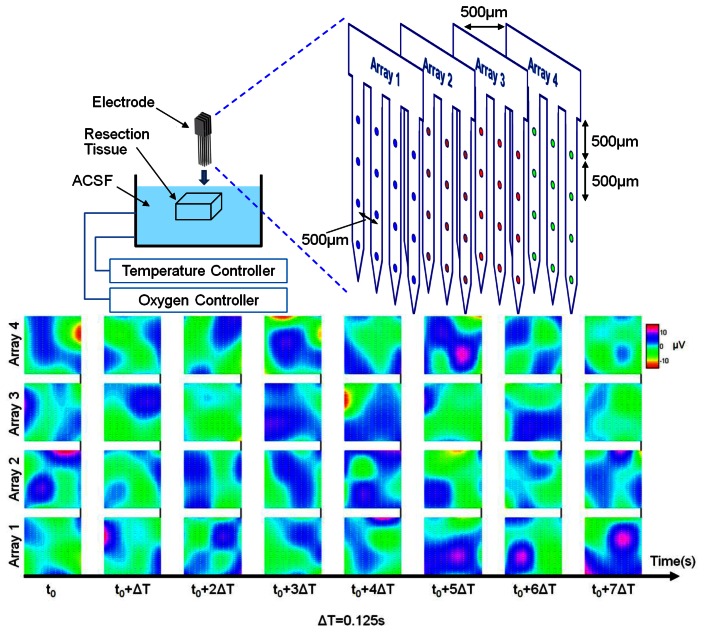
3D distributed near-field potential recording by 3D probe array from cerebral resection cortex tissue.

Next, real 3D neural signal propagation observation in human cerebral resection cortex layers is achieved by practical implantation after resection surgery in cooperation with the Buddhist Tzu-Chi Hospital in Hua-Lien City, Taiwan. All the study procedures follow the IRB rule of Buddhist Tzu-Chi Hospital. A patient suffering from epilepsy seizures needed surgery resection treatment. After the resection surgery, the tissue was put into a prepared artificial cerebrospinal fluid (ACSF) solution in a water bath with 37 °C temperature control and oxygen supply to keep it alive. Usually the neural activity in resection tissue can last for about 2 hour in the solution. The fabricated 3D probe array was inserted into the brain tissue by a manual 3-axis moving stage. From the recording, we can build a 3D distributed spatial signal plot. Figure 10 illustrates the reconstructed 3D near-field potential distribution recorded by 3D probe array in brain, which allows further studies for event-related observation.

**Figure 11.**
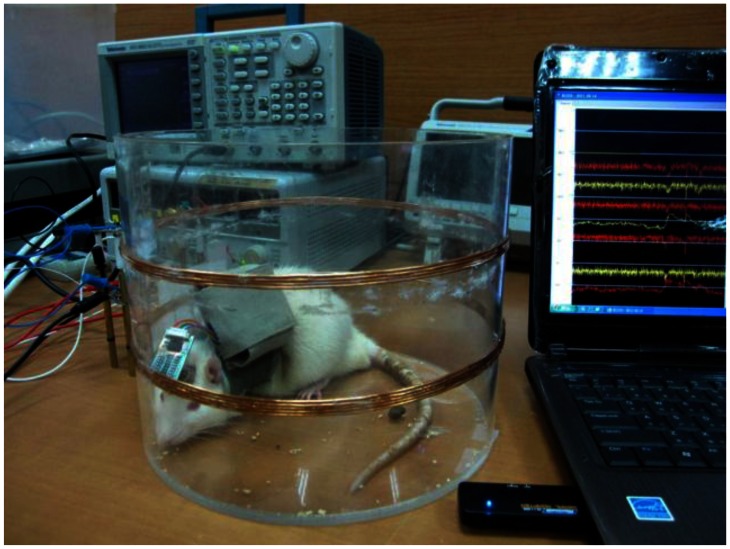
Induced seizures recording on rat by the present microsystem.

Finally, the present microsystem platform was applied to the recording of pentylenetetrazole (PZT)-induced seizures in rat [27]. Figure 11 shows the photograph of the environment setup. The main board is carried by the backpack on the rat. RF power is generated by a function generator and sent into a customized power amplifier. Power transmission coils connected to the power amplifier are located on the walls of the cage. Recorded data is transferred to a laptop via Bluetooth transmission. Customized Windows-based software provides continuous monitoring and data storage on the laptop.

Sprague Dawley rats aged 10–12 weeks and with 500–800 g body weight were used in the experiments. Pentylenetetrazole (PTZ) was prepared fresh daily, dissolved in 0.9% saline.

**Figure 12.**
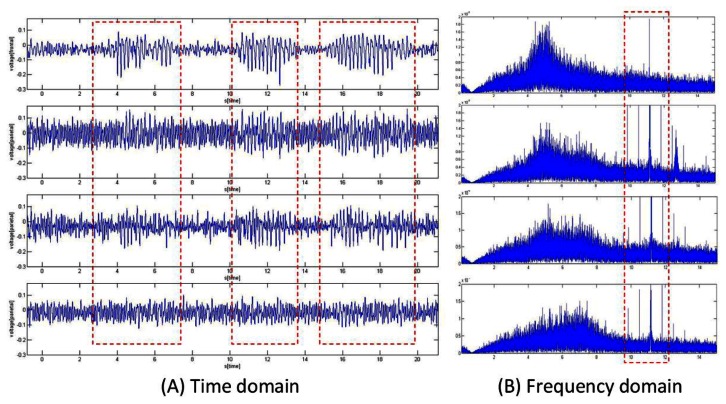
Recorded spike-wave (SWD) discharge distributions from frontal to parietal (**A**) Time domain plot (**B**) Frequency domain plot. Red dotted rectangle denotes the SWD part founded in the recording.

After the microsystem was attached to the rat, PZT was administered intraperitoneally in a volume of 10 mL/kg body weight. Figure 12 shows the selected four channels located from frontal to parietal region of the cortex. Red dotted rectangles denote the SWD part found in the recording.

From Figure 12(A), it is clear that significant SWD can be found in the frontal zone, and its magnitude is decreased as the recording location is further from the frontal due to the fact that PZT-induced seizures mainly occur in the frontal. Figure 12(B) illustrates the frequency analysis of the recorded data, obvious peaks can be observed in the frequency range of 8–12 Hz which is the main seizure component range.

## 6. Discussion and Conclusions

In this paper we present a wireless and battery-less system platform and demonstrate it in various implantation applications with different neural sensors. The main contribution of this paper is to integrate different parts, including sensors, customer-designed circuits and commercial chips, into a complete system for neural sensing. The complete system weights 13.17 g, which meets the requirements for being readily carried by rats [28]. Different sensing targets, including ECoG and near-field neural activities, can be monitored by the proposed grid electrode array and 3D array. Each part of the system is designed and characterized in the paper. Practical *in vivo* recordings including auditory response, brain resection tissue and PZT-induced seizure recordings demonstrate the correct functioning of the proposed system. Currently, some similar works using analog wireless transmission techniques [28,29] are reported for neural sensing applications. In this case, these systems can provide small packaging and lightweight without digital processing circuits, but they may also easily suffer interference from surrounding noise. Highly integrated systems also have been reported for small flying insects [30], hence, to integrate the circuits into monolithic chip will be our next step. Two kinds of electrode design are reported in this paper for measurement on brain surface and in brain tissue. It will be another issue to integrate them into a single sensor to provide observation on and in the brain at the same time.

## Figures and Tables

**Table 1.** Summary of measured specifications.

**Head tage**	16-CH Amplifier	Gain	Tunable 52–75 dB
		Band	Tunable 0.1–6 KHz
		CMRR	>90 dB
		Current	4.5 μA per channel
		Supply Voltage	1.8 V
		PSRR	78 dB
		Input referred noise	2 μVrms
		Chip area	4.18 mm^2^
	Weight		3.92 g
	Size		1.8 × 3.4 cm^2^
**Main Board**	Low Dropout Regulator	Output Voltage	1.8–3.3 V
		Line Regulation	4.5 mV
		Load Regulation	5 mV
		Current Efficiency	99%
		Output Current	0∼200 mA
		Quiescent Current	45 μA
		Current Limit	252 mA
		Temperature Raise	<2 °C@200 mA
		PSRR	71 dB@10K Hz
	MCU	ADC Resolution	12 bit
		Sample Rate	1.25 kHz per channel
		Current	200 μA
		Supply Voltage	1.8 V
	Blue-tooth	Transmission rate	Max 961,200 bps
		Current	15 mA
		Supply Voltage	1.8 V
	Weight		9.35 g
	Size		3.9 × 3.9 cm^2^
**Overall System**	Weight		13.17 g
	Current Consumption		<20 mA
